# Hydrophobicity and Biodegradability of Silane-Treated Nanocellulose in Biopolymer for High-Grade Packaging Applications

**DOI:** 10.3390/polym14194147

**Published:** 2022-10-03

**Authors:** Indra Surya, C. M. Hazwan, H. P. S. Abdul Khalil, Esam Bashir Yahya, A. B. Suriani, Mohammed Danish, Azmi Mohamed

**Affiliations:** 1 Department of Chemical Engineering, Universitas Sumatera Utara, Medan 20155, Indonesia; 2 Bioresource Technology Division, School of Industrial Technology, Universiti Sains Malaysia, Penang 11800, Malaysia; 3 Nanotechnology Research Centre, Faculty of Science and Mathematics, Universiti Pendidikan Sultan Idris, Tanjong Malim 35900, Perak, Malaysia; 4 Bioprocess Technology Division, School of Industrial Technology, Universiti Sains Malaysia, Penang 11800, Malaysia; 5 Green Biopolymer, Coatings and Packaging Cluster, School of Industrial Technology, Universiti Sains Malaysia, Penang 11800, Malaysia

**Keywords:** seaweed, kenaf cellulose nanofiber, silane surface treatment, biopolymer film, edible film, high-grade packaging

## Abstract

The growing concern about pollution produced by plastic waste and the consequent environmental dangers has led to increased interest in replacing plastics with sustainable and biodegradable alternatives. Biopolymers such as seaweed have been examined for their film-forming characteristics to make edible films for packaging applications. This study aimed to prepare biopolymeric packaging films through a solvent-casting process using natural red seaweed (Kappaphycus alvarezii) and kenaf cellulose nanofiber (CNF), followed by film surface treatment using silane. The hydrophobic properties of the seaweed/CNF biopolymer were examined through water solubility (WS), moisture absorption capacity (MAC), water vapor permeability (WVP), and contact angle (CA) measurements. Fourier transform infra-red (FT-IR) film spectra clearly showed successful modification of the seaweed film (SF) by silane and the incorporation of kenaf CNF over the surface of the seaweed film. The wettability-related analysis showed positive results in determining the modified film’s hydrophobicity properties. Film degradation analysis using the soil burial method showed a lower degradation rate for films with a higher CNF loading. Overall, the characterization results of the seaweed/CNF biopolymer film predicted hydrophobicity properties. The slow degradation rate was improved with surface modification using silane treatment and the incorporation of kenaf CNF filler with the seaweed matrix. As a result, we found that the seaweed/CNF biopolymer film could be used as high-grade packaging material in many potential applications.

## 1. Introduction

Concerning severe environmental adverse effects, notably pollution, it is critical to replace traditional petrochemical plastics with the most effective, eco-friendly, and biodegradable materials available. There is a growing emphasis on developing bio-based products and innovative processing methods that contribute to sustainability and decreased reliance on fossil fuels [1]. The environmental issues caused by finite resources and the continued rise in the pricing of petroleum-based polymers have been of great concern to researchers [2,3]. Moreover, fossil fuel combustion and the disposal of non-degradable and toxic petroleum-based plastics significantly impact climate change [4]. Therefore, recently, the focus of research on the preparation and characterization of packaging films has shifted from petroleum-derived plastics to renewable and environmentally friendly biopolymer films [5].

Seaweed is a low-cost source of polysaccharide biomass and is abundant, sustainable, and environmentally friendly. It is an important source of natural biopolymer, which is widely utilized across the world. Because of its outstanding phycocolloid content, biodegradability, high accessibility, and high organic content, seaweed derivatives such as alginate and carrageenan have mostly been used in agriculture, cosmetics, packaging, medicines, and food [6]. Carrageenan is a natural polymer that is derived from edible red seaweeds of the Rhodophyceae family. It has been used as a thickener or gelling agent in a variety of sectors, including food, pharmaceuticals, and other industries [7]. Carrageenan has great potential as a gel-forming material since it can produce a gel via ionotropic gelation, along with a method that involves helix formation during cooling and crosslinking in the presence of potassium or calcium ions [8].

Packaging materials comprised of edible components are known as edible films. Because of their numerous advantages, biopolymer films made from renewable resources are now widely utilized as packaging materials [9]. This is because edible films can lengthen the quality, freshness, and shelf life of packed products. The edible films cover the packed product to form a semipermeable barrier, increasing its barrier characteristics by reducing moisture, lipids, gases, and volatile exchange [10]. There are a few disadvantages to using natural polymers such as carrageenan and cellulose. Due to their hydrophilic nature, these natural materials are only suitable for a limited range of applications [11]. Biopolymers must possess physicochemical characteristics that are equivalent to those of synthetic polymers in order to be considered viable prospective substitutes for synthetic polymers in the marketplace [12,13]. Therefore, the current study was carried out using chemical modification of a silane surface treatment to improve the films’ performance, especially in terms of their hydrophobicity. Unlike most additives, which have a restricted performance range, silanes can produce hydrophobic to hydrophilic surface characteristics. Adhesive bonding may occur as a result of hydrophobic contact. Simultaneously, self-cleaning surfaces are often coupled with extreme hydrophobicity [14].

Nanocellulose as a reinforcing material in nanocomposites is a potential field of investigation. It is an abundantly available and renewable material with low density, high specific properties, biodegradability, and acceptable surface reactivity. The properties of materials further improve when nanocellulose is used as a reinforcing phase in the nanocomposite. The performance of nanocellulose-reinforced nanocomposites is better than micro- or macro-cellulose composites. The design flexibility and easy processability of nanocellulose/polymer increased its demand in various industrial sectors, including automotive, packaging, electronics, and biotechnology [15]. It also has reactive hydroxyl groups, which makes it excellent for surface functionalization in a wide range of applications [16]. Natural cellulosic fibers, such as kenaf, have the potential to be used in polymeric composites in stead of synthetic fibers. Kenaf is mostly made up of cellulose (about 70%), which explains its superior mechanical properties. Because of its superior mechanical performance, cellulose has gained increased interest and attention as reinforcement in biodegradable polymer films. It is generally acknowledged that incorporating cellulose into a biodegradable polymer film could be an efficient strategy to overcome the poor properties of the biodegradable polymer while obtaining desired function and features [17]. Developing biopolymeric films from seaweed and kenaf CNF is a strategy to increase the value of biodegradable packaging materials while promoting the use of low-cost sources of these polysaccharides. In this work, the potential of CNF as a filler in a seaweed polymer film, as well as the effectiveness of silane-modified film surfaces in enhancing the hydrophobicity characteristics of the polymer matrix and improving film stability during degradation have been further explored.

## 2. Materials and Methods

### 2.1. Materials

The red seaweed (Kappaphycus alvarezii) used in this project was supplied by Green Leaf Synergy Sdn. Bhd. CNF was extracted from Kenaf bast and utilized as nanofiber reinforcement with a seaweed matrix. The bast fibers were extracted from the stems using a Sprout-Bauer refiner (USA), model number: R34EM, Fitchburg, MA, USA. ZARM Scientific & Supplies Bukit Mertajam, Penang, Malaysia supplied the SC-CO_2_ (Malaysia). All chemicals, including triethoxymethylsilane ((C_2_H_5_O)3SiCH_3_), sodium chlorite (NaClO_2_), hydrogen peroxide (H_2_O_2_), acetic acid (CH_3_COOH), and sodium hydroxide (NaOH), were provided by Sigma-Aldrich, Subang Jaya, Selangor, Malaysia. These chemicals were analytical grade.

### 2.2. Isolation and Characterization of CNF via Supercritical CO_2_

A total of 500 g dry weight of raw kenaf bast fibers were heated at 160 °C for 3.5 h in an alkaline medium containing 25% by weight of NaOH and 0.2 wt% of anthraquinone (AQ) to produce alkali-treated fibers. These fibers were then washed in deionized water to remove surface impurities and to also remove degraded hemicellulose and other extractives. The next step was a bleaching procedure using 3% H_2_O_2_ and 0.5% MgSO_4_ at 80 °C for two hours. To produce CNF using the SC-CO_2_ method, bleached fibers were exposed to supercritical carbon dioxide (SC-CO_2_) at 60 °C for two hours. A total of 5% acetic acid was added to the CNF in the final stage of the process to complete the isolation process. The seaweed biofilm was reinforced with CNF that was extracted using the SC-CO_2_ method.

### 2.3. Preparation of Macroalgae/CNF Biopolymer Film

With the addition of various amounts of CNF filler, 10 g of raw macroalgae and 5 g of glycerol were soaked in 500 mL of deionized water before cooking at 85 °C with continuous stirring to create a suspension. The macroalgae/CNF biopolymer films used in the study were plasticized using glycerol. CNF was added to the macroalgae biopolymer films at percentage concentrations of 1%, 2%, 3%, 4%, and 5%. After casting the hot solution onto the plate and transferring it into the oven at 45 °C for 24 h, the dried macroalgae biopolymer film was produced. The oven-dried macroalgae biopolymer films were desiccated for two days at a relative humidity of 50% before being examined and analyzed as test samples.

### 2.4. Surface Treatment of Macroalgae Film Using Silane

Triethoxymethylsilane was used in the chemical modification procedure, which was carried out in a fume chamber. A mixture of acetone with 40% silane solution was used to modify the surface of the biopolymer films. The prepared silane solution was added to the reaction flask along with the seaweed/CNF biopolymer films. To start the condensation process, a reaction flask was connected to a reflux condenser with a steady water flow. The reaction flasks with reflux condensers were submerged in silicone oil. Surface treatment was carried out at 90 °C for three hours. The macroalgae/CNF biopolymer films were placed and cleaned inside beakers using acetone to eliminate excess silane after modification. The resulting films were then vacuum-dried for three hours at 70 °C. The films were then dried in an oven for 24 h at 40 °C.

### 2.5. Characterization of Seaweed/CNF Biopolymer Film

#### 2.5.1. Particle Size and Zeta Potential

The particle size and zeta potential of the extracted CNF was calculated via the dynamic light scattering (DLS) method using a Malvern Zetasizer Nano ZS 7.11 instrument (Malvern Instruments Ltd., Malvern, UK). After adding distilled water with a refractive index of 1.330 as a dispersant, the sample was sonicated for 10 min. The measurement of pH of each suspension was adjusted by adding NaOH or HCl while using predetermined parameters, including a viscosity of 0.8872 and material refractive indices of 1.47. The zeta potential of the original aqueous CNF suspension in a 0.1 mM KCl electrolyte was calculated as a function of the concentration of the suspension.

#### 2.5.2. FT-IR Characterization

The chemical functional groups in the Seaweed/CNF composite film were determined using Fourier transform infrared spectroscopy (PerkinElmer, PC1600, USA), and the transmission spectra of composite films were recorded using the attenuated total reflectance (ATR) method in the wavenumber range of 400–4000 cm^−1^. Film samples were prepared by cutting them into (3 × 3 cm^2^) and oven-drying them at 60 °C for 24 h prior to FT-IR analysis.

#### 2.5.3. Water Solubility (WS)

The solubility test was conducted following the Romero-Bastida et al. (2005) method with some modifications. The Solubility of the film (S%) was calculated based on the percentage of the dry film matter (3 × 3 cm^2^) that had solubilized after 30 min of immersion in 80 mL of deionized water with constant agitation at room temperature (25 °C). The remaining piece of film was then filtered using filter paper (Whatman No. 1) and dried inside the oven at 60 °C until it achieved a constant weight. The film solubility was calculated as a percentage of the soluble carbohydrate to the initial solid weight of the film.

WS (%) = [(DM_0_ − DM_30_)/DM_0_] × 100 (1)

The WS was calculated using Equation (1), where DM_0_ refers to the first dried material (before the solubilization) and DM_30_ refers to the dried material after 30 min of solubilization.

#### 2.5.4. Moisture Absorption Capacity (MAC)

The moisture absorption of the seaweed/CNF composite film samples was determined based on the Ghanbarzadeh and Almasi (2011) method following Equation (2). To test film moisture absorption, a 30 mm × 30 mm film was initially dried at 100 °C in an oven until a constant weight (W_1_). The film samples were weighed, and then, left at room temperature for 48 h in a desiccator with distilled water (100% RH). After 48 h, the conditioned sample was removed from the desiccator and immediately weighed (W_2_). All measurements were carried out in triplicate.

MAC (%) = [(W_2_ − W_1_)/W_1_] × 100 (2)

#### 2.5.5. Water Vapor Permeability (WVP)

The water vapor permeability test was conducted using the ASTM E96-00 method with some modifications. Circular test cups were tightly covered with seaweed films and sealed with silicone grease. The film samples were cut into round shapes and kept at the same size as the lid of the cup, which was around 43 cm². A total of 50 mL of distilled water was used to fill the test cup before the film was attached to it. The film thickness and initial weight for each test cup were initially recorded. The test cup was then placed in the weathering chamber for 6 h (50% RH, 25 °C). The weights were increased every 1 h and continued to increase for the next 6 h. Linear regression was used to determine the slope of the straight line at various points (R^2^ > 0.99). The following equations were used to determine WVP (g mm/m^2^d kPa) and WVTR (g/m^2^d):

WVTR = (slope/A) = [(Δm/Δt) × A](3)

WVP = (WVTR × X)/ΔP(4)

Based on Equations (3) and (4), Δm/Δt is the weight of moisture gain per unit of time (g/d), ‘A’ is the area of the exposed film surface (m^2^), ‘X’ is the film thickness (mm), and ΔP is the difference in partial pressure (kPa).

#### 2.5.6. Contact Angle (CA)

The contact angle (CA) of water on the film surface was measured using a sessile drop technique using a CA analyzer (KSV CAM 101) at room temperature after a drop of 6 µL was placed on the film surfaces using a microsyringe that was operated automatically. Initially, all the film samples on the movable sample stage were leveled horizontally prior to measurement. The CA was measured on both sides (left and right) of the drop, and their values were added. For each film sample, five measurements were taken and their results were averaged.

#### 2.5.7. Soil Burial Testing

The soil burial test of seaweed/CNF composite films was performed in a plastic container with a capacity of 1000 mL and above. The soil samples used were high-quality dark garden-soil samples with a moisture content (MC) of 35.2 ± 0.35%. Each film sample (30 mm × 30 mm) was weighed to measure its initial weight (Mo) before being buried in a container with 5 cm of soil that was between 30 and 50% humidity at room temperature. Water was put in the soil once every 2 to 3 days to maintain soil moisture and for the microorganisms to be active.

At different time intervals (7 days, 14 days, 21 days, and 28 days), film samples were removed from the container, gently cleaned and brushed, dried at 50 °C for 6 h until a constant weight, and kept in a desiccator at 50% RH in order to settle the temperature before weighing. Based on Equation (5), which uses M_o_ as the initial mass of the films prior to the test and M_f_ as the residue mass of the films following the test, the percentage weight loss of the samples was calculated.

Weight loss (%) = [(M_o_ − M_f_)/M_o_] × 100 (5)

## 3. Results and Discussion

### 3.1. Zeta Potential and Particle Size of Kenaf CNF

**Figure 1:**
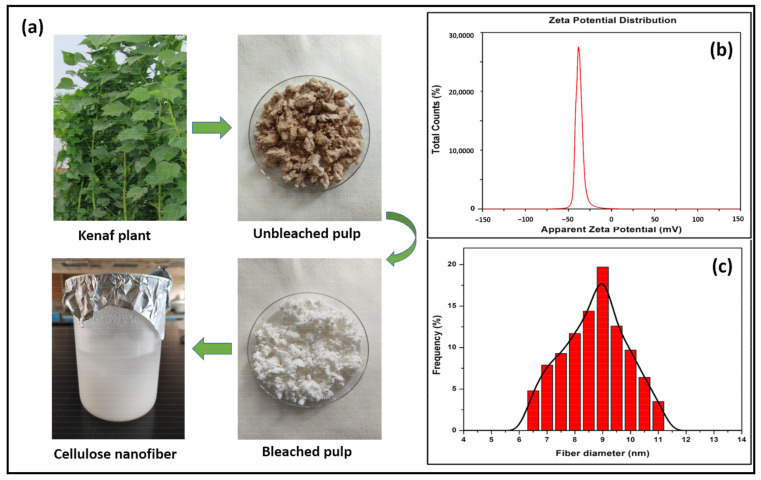
Material transformation (**a**), zeta potential value (**b**) and particle size distribution (**c**) of kenaf CNF.

The zeta potential measurement was used to investigate the colloidal stability of the CNF dispersion in aqueous media. The zeta potential value of the kenaf CNF is shown in Figure 1b. Higher zeta potential values represent a higher capacity for dispersion, while low values indicate low dispersion stability [18]. The zeta potential of the kenaf CNF was found to be stable because the absolute value should be greater than −25 mV with no propensity to flocculate [19]. This number indicates that sufficient mutual repulsion occurred, resulting in colloidal stability, which may be a necessary condition to improve the mechanical characteristics of nanocellulose-based composites [20].

The zeta potential (estimated as the surface charge) may be determined by observing the rate at which negatively or positively charged particles rise when exposed to an electric field. The isolated CNF suspension exhibited a strong negative value of −32.0 mV, generated by the SC-CO_2_ treatment in conjunction with mild hydrolysis of oxalic acid. The extracted CNFs had a high negative zeta potential due to the presence of oxalate groups generated during moderate oxalic acid hydrolysis. In general, cellulosic surfaces are bipolar, with a predominant acidic contribution due to the proton in the hydroxyl functional group and the carboxyl groups present from oxalic acid [20].

The CNF was nanoscale in size; produced from the kenaf pulp through the SC-CO_2_ extraction method, it exhibited a smaller and more homogeneous size distribution, which is attributed to the microfibrils bursting into nanofibrils, as shown in Figure 1a. Figure 1c depicts the size distribution of the kenaf CNF. The size distribution of the kenaf CNF was in the range of 6–12 nm. The maximal average diameter of the kenaf CNF was measured to be 9 nm. The kenaf CNF was homogenized at a continuous pressure of 50 MPa in a high-pressure homogenizer throughout the extraction process to aid in the defibrillation of cellulose fiber. The SC-CO_2_-extracted CNF exhibited narrow intensity and a smaller size; this indicated the effectiveness of the defibrillation procedure, prior to further isolation of the CNF, in helping to produce a more homogeneous and stable CNF. However, for further mild hydrolysis of oxalic acid, high pressure was applied to increase the surface area of the substrate by disrupting the secondary interactions between the fibers and defibrillating materials [21]. It is an effective method to extract CNFs from kenaf fibers. The lignocellulosic structure has been assumed to disintegrate chemically during the SC-CO_2_ method [22]. According to a zeta potential analysis of a kenaf CNF and its particle size distribution, the particle stability behavior of the kenaf CNF was classified as stable with a homogeneous particle size distribution [23].

### 3.2. FT-IR Characterization of Modified Seaweed Film

**Figure 2:**
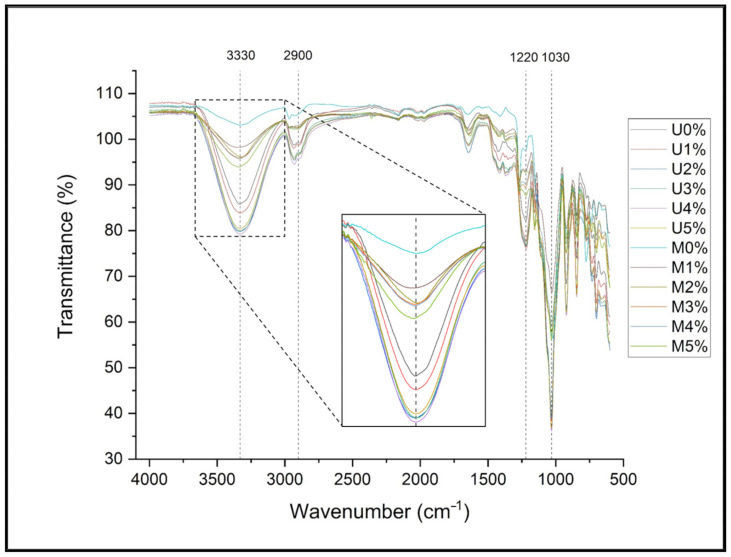
Overlapring FT-IR spectra of seaweed/CNF composite film.

**Figure 3:**
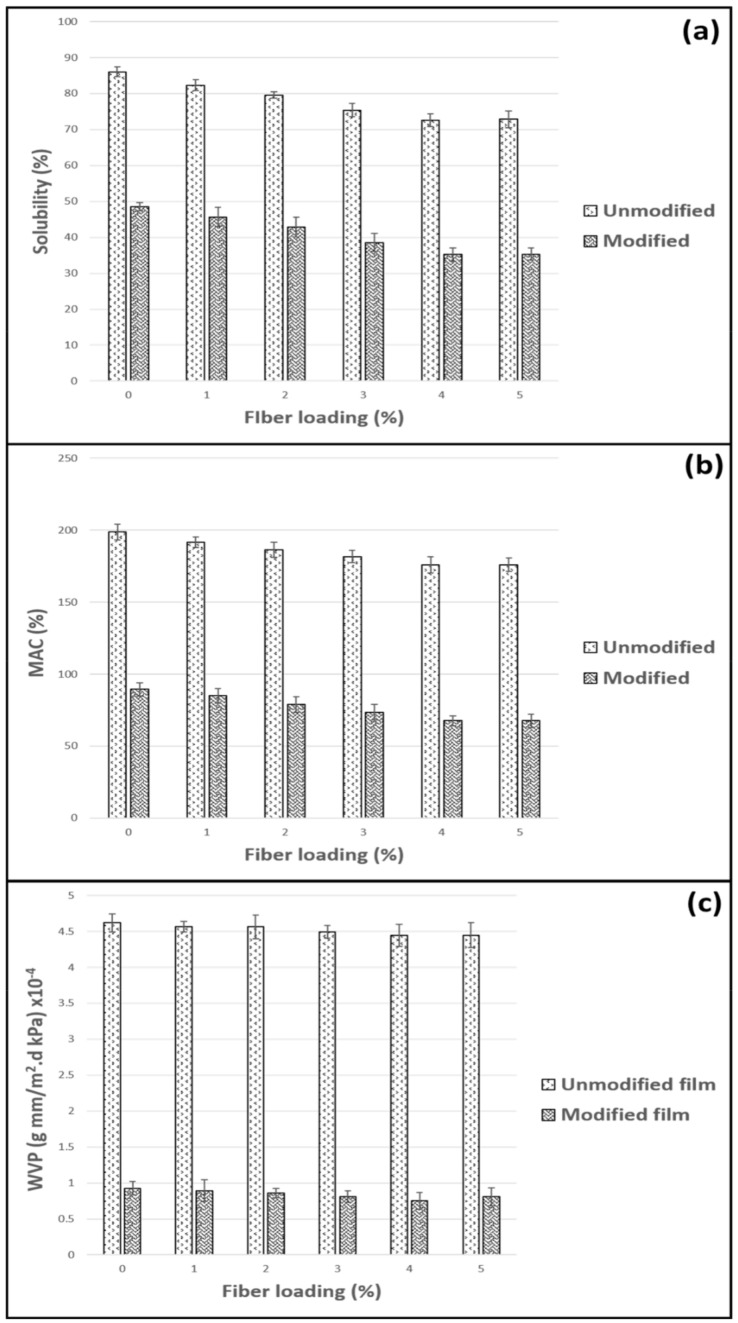
Water solubility (**a**), moisture absorption capacity (**b**), and water vapor permeability (**c**) of modified and unmodified seaweed/CNF composite film.

FT-IR is the most frequent approach for identifying chemical changes in materials by differentiating the presence and absence of functional groups in compounds. Figure 2 and Figure 3 show the Fourier transform infrared spectroscopy (FT-IR) spectra of the unmodified and silane-modified seaweed/CNF composite films and their functional groups. There was a slight difference in terms of the FT-IR result, such as the intensity value, peak broadening, and presence of a new absorption peak after the silane treatment process, compared to that of the unmodified SF. The differences in the FT-IR trend for each modified SF sample also seem to have been effected by the amount of CNF filler loading into the SF.

The overlap variations in the FTIR spectra of the unmodified and silane-modified SF are clearly seen in Figure 2. The presence of characteristic silane peaks in the FT-IT spectra confirmed its effective modification. The typical vibrations of CH3 in silane were attributed to the bands at 775 cm^−1^ (CH–H3) and 2980 cm^−1^ (C–H) [24]. Furthermore, Si–CH_3_ bending vibrations with a frequency of 1250 cm^−1^ were also identified from the FT-IR result [25]. It should be noted that after silane surface modification, the peak of intensity of the hydroxyl group absorption (OH) at around 3330 cm^−1^ decreases dramatically due to the removal of numerous hydroxyl groups through the interaction with the silane agent [26]. A large intensity gap was observed at this absorption peak between the unmodified and modified SFs.

Following the addition of CNF to the seaweed film matrix (SF), certain changes may be seen. As the CNF was incorporated into the SF, the intensity and width of the overall OH band increased considerably, as presented in Figure 2. This could indicate that the hydrogen bonding between the seaweed (carrageenan) and CNF increased [27]. Peaks were observed at 1217–1220 cm^−1^ in all composite films, and were caused by the kappa-carrageenan stretching of the sulphate group (S=O) in seaweed [28]. All types of carrageenan had asymmetric stretching vibrations of the S=O bond in the range of 1210 to 1270 cm^−1^ [29]. Finally, the sharp peaks at 1031 cm^−1^ and 844–912 cm^−1^ might be due to the glycosidic linkage (C-O) of 3,6-anhydro-D-galactose, the stretching of C-O-S in (1-3)-D-galactose, and the stretching of C-O-C in 3,6-anhydrogalactose, all of which are commonly associated with the presence of carrageenan in seaweed films [30].

### 3.3. Physical Properties

Several physical analyses were conducted to test the performance of the seaweed/CNF film with the interaction of moisture. This analysis was necessary to determine the effect of silane surface treatment on film hydrophobicity properties. Figure 3 shows the water solubility (a), moisture absorption capacity (b), and water vapor permeability (c) of the seaweed/CNF composite film. Both modified and unmodified films with different fiber loadings were studied to determine the effect of CNF loading (%) on the WS of the composite film. Figure 3a shows the water solubility (WS) of the modified and unmodified seaweed/CNF composite films. The unmodified seaweed/CNF composite film had the highest overall value of WS compared to the silane modified seaweed/CNF composite film. The neat unmodified SF had a WS value of 86.1%, marking the highest rate of solubility among all the samples tested. Regarding the silane-modified SF, the neat film had a lower value of WS at 48.5%, which was 44% lower compared to the unmodified neat SF. With the addition of CNF to the film, the values of WS for both the unmodified and modified films were reduced to some extent. The composite film with 1% CNF loading had WS values of 82.3% and 45.7% for the unmodified and modified films. The WS values of the film decreased down to 72.5% and 35.2% at a 4% CNF loading for the unmodified and modified seaweed/CNF composite films. Slightly higher results of WS were observed at 5% CNF loading for both the unmodified and modified films, but the values were still lower than those of the film at 3% CNF loading. The addition of CNF to the SF decreased the WS rate for both films. The film with 4% CNF loading had around a 16% lower WS rate compared to neat SF.

Seaweed films with a lower WS rate are more resistant to water. In comparison to unmodified SF, the interactions between water and modified SF were reduced as a result of silane surface treatment. The difference is due to the fact that silane-modified SF contains lower hydroxyl group (OH) levels than unmodified SF. The efficient reduction of hydroxyl groups through surface silane treatment is a major component in reducing the interaction between water and the SF. Following silane modification, polysiloxane particles developed on the surface of the SF, resulting in hydrophobicity in the fabricated silane-modified SF [24]. The reduction in WS of seaweed composite films, caused by the introduction of CNF at various loading concentrations, was attributed to the establishment of strong intermolecular hydrogen bonds between the CNF fillers and the seaweed matrix. This method of formation gradually lowered the available hydroxyl groups in seaweed and reduced the solubility rate of the seaweed composite films in water. The surface modification of silane and the addition of CNF to the SF were able to minimize the rate of water solubility of the films to some extent by reducing the water interaction with the film when compared to neat seaweed films. In another study, CNF incorporated into whey protein isolate-based films was reported to reduce moisture content and water solubility [31].

Figure 3b shows the moisture absorption capacity of the unmodified and modified seaweed/CNF composite films with different percentages of CNF filler. Unmodified SF had a higher overall value of MAC compared to modified SF for the SF sample tested. The neat SF with 0% CNF filler loading had the highest value of MAC recorded at 198.8%. As for the neat modified SF, the MAC value was found to be 89.3%, which is 45% lower compared to unmodified SF. The reduction in the MAC values of both the unmodified and modified films was observed with the addition of CNF filler loading into the SF. At 1% CNF loading, the MAC value of the unmodified film was reduced to 191.7% which is 3.5% lower compared to the neat unmodified film. The modified SF also showed an improvement in the MAC value with the addition of 1% CNF loading, with a 4.9% reduction in MAC compared to the neat modified film. The MAC values were reduced to a minimum of 175.1% and 67.5% for the unmodified and modified seaweed composite film, respectively, at 4% CNF loading. No improvement was made at 5% CNF loading. The MAC values were slightly higher than the film with 4% CNF; however, this is still much lower compared to the previous SF with 3% CNF loading tested.

The reduction of the hydroxyl group (OH) by the silane surface treatment was attributed to the reduction in the MAC value for all of the modified SFs. The silanols produced via silane hydrolysis reacted with the surface hydroxyl groups of the seaweed film. The interaction of silanols with hydroxyl groups produced a covalently bonded silane layer on the surface of the SF and the creation of polysiloxane particles was attributed to the self-polymerization [25]. The reduction in the MAC of the films, caused by incorporation of CNF, could be attributed to the CNF in the network structure of the film matrix, which reduces the accessible space in biopolymer for the placement and interaction of water molecules [32]. Furthermore, their comparable chemistry may have resulted in good interface adhesion, which could prevent moisture absorption to some extent [33]. Strong intermolecular hydrogen bonding between the hydroxyl groups of the CNF and the OH-seaweed groups limited the flexibility of the seaweed molecules, lowering the MAC of the seaweed films. As a result, the addition of CNF enhanced the water sensitivity of the seaweed composite films.

The water vapor permeability (WVP) of the unmodified and modified seaweed/CNF composite films was determined using the gravimetric technique. Figure 3c shows the WVP for the unmodified and silane-modified SF samples with varying CNF filler loading. The WVP of the silane-modified seaweed/CNF composite films was greatly improved compared to the unmodified films for neat film, as well as films with CNF loading. At 0% CNF loading, the WVP value for unmodified and modified SF was recorded at 4.62 × 10^−4^ g mm/m^2^.d kPa and 0.92 × 10^−4^ g mm/m^2^.d kPa, respectively. The modification of neat SF by silane reduced the WVP value by 80% compared to the unmodified film. The addition of CNF filler loading into the SF reduced the WVP value somewhat. At 1% CNF incorporation, the WVP values for unmodified and modified film were found to be 4.56 × 10^−4^ g mm/m^2^.d kPa and 0.89 × 10^−4^ g mm/m^2^.d kPa, respectively. The addition of CNF to the SF at 1% loading reduced the WVP value by around 1.23% compared to the neat SF with 0% CNF loading for both the unmodified and modified SF. The improvement of the WVP with the incorporation of CNF was relatively low, but reducing the WVP value of the SF would be of great benefit for the films’ applications. A total of 4% CNF loading resulted in the lowest WVP values at 4.44 × 10^−4^ g mm/m^2^.d kPa and 0.75 × 10^−4^ g mm/m^2^.d kPa for the unmodified and modified seaweed/CNF composite films, respectively. Adding 4% CNF filler loading to the SF decreased its WVP value by around 3.84% compared to the neat SF with 0% CNF loading for both the unmodified and modified SF. No improvement was made at 5% CNF loading for either unmodified or modified film. The WVP value at 5% CNF loading for both film groups shows an almost identical value to the previous 4% CNF loading. The WVP values at 5% CNF loading were slightly increased compared to the previous 4% CNF load. The value, however, was still relatively lower compared to WVP at 3% CNF loading for both the unmodified and modified SF.

The principle of having a lower WVP rating was closely tied to the WS and MAC tests, which was previously explained. The hydroxyl group of the film membranes was greatly decreased by the silane-based SF treatment. When hydrolyzed silane solutions are combined with SF, reactive silanol groups have a strong attraction to each other, creating Si-O-Si bonds, as well as hydrogen connections with the hydroxyl sites of fibers. The surface treatment resulted in the incorporation of a protective hydrophobic layer of polysiloxane into the film, enhancing its resistance to moisture. In general, silane surface treatment showed the capacity to improve the water barrier qualities of seaweed films by reducing their water vapor permeability (WVP) values. WVPs were also reduced due to the dispersion of impermeable crystalline nanocellulose particles in the matrix, which created a tortuous path, and therefore increased the effective diffusion and pass length for those water molecules [34,35]. Small particles would increase the tortuosity patch and make it difficult for the steam to pass through the film.

### 3.4. Contact Angle (CA)

Contact angle measurement is the most widely used method for predicting hydrophilic/hydrophobic surfaces. It is established that for hydrophilic surfaces, the water contact angle should be less than 90°; for hydrophobic surfaces, the contact angle should be greater than 90° and less than 150°; and for super-hydrophobic surfaces, the contact angle should be greater than 150° [36]. The CAs of the unmodified and modified seaweed/CNF nanocomposite films are shown in Table 1. The contact angle values were strongly affected by the silane modification of the films. The unmodified control film with a 0% CNF load had the lowest value of CA compared to the rest of the SFs tested, at 39.945 ± 0.26°. For the modified control film, the CA was recorded at 107.27 ± 0.29°, which is 62.77% higher compared to the unmodified control film. SFs that wenr through the silane modification process had higher overall values compared to the unmodified SFs. The addition of CNF filler to the SF also improved the CA. At 1% CNF loading, the CA for the unmodified and modified films was found to be 41.35 ± 0.50° and 109.07 ± 0.27°, respectively. Adding 1% CNF filler increased the CA value by approximately 3.5% compared to neat SF with 0% CNF loading for both the modified and unmodified films. The highest recorded CA value of all the SF samples tested was found to be 117.73 ± 0.91° for the modified film with 4% CNF filler loading. An improvement of 9.75% of the CA value was made at this point compared to the neat modified film. Increasing the CNF content to 5% did not show any significant improvement in the CA value. Instead, the CA values were slightly lower than the film at 4% CNF loading, but still higher than the film with 3% fiber loading. This behavior occurred for both the unmodified and modified films.

The lower value of the contact angle is due to the natural properties of seaweed, which is highly hydrophilic. In comparison to neat SF, silane-modified SF exhibited significantly improved hydrophobicity properties. The hydrolysis of silane and the subsequent interaction with additional silanols or isolated hydroxyl groups on the surface of the SF can be attributed to the formation mechanism of the treated SF. In this study, the silane-modified SF had a much higher water CA value than the control film. This indicates that the polysiloxane deposited on the surface of the SF increased the hydrophobicity of the SF [37]. CNF hydrogen bonds to the seaweed hydroxyl group and reduces the number of free hydroxyl groups of the seaweed film, which increases the contact angle due to a decrease in water affinity [32,38]. The increased intermolecular hydrogen bonding between the OH groups of the seaweed and CNF contributed to the improvement in the CA of the seaweed films with the addition of CNF fillers. As a result of this interactions, the interaction of the water and film decreased, resulting in a reduction in the hydrophilicity of the films. The improvement in the hydrophobicity of the film surfaces could also be attributed to the increased surface roughness of the films due to the incorporation of fillers [39].

**Figure 4:**
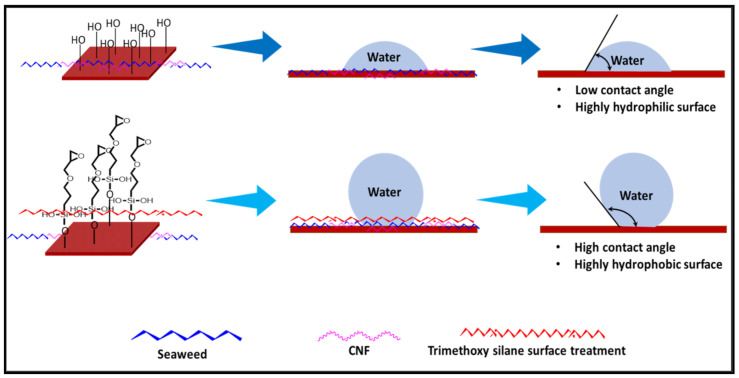
Silane modification effect on seaweed film contact angle.

An SF that has a high CA value would have better resistance to moisture and water. In packaging applications, this would offer huge advantages to variations in use and also offer good protection in wet conditions. Figure 4 shows the effect of silane modification on the contact angle of seaweed films. Having a high CA value is crucial for the barrier properties of a SF. The silane-modified SF had greatly improved hydrophobicity compared to unmodified SF. The formation mechanism for the treated SF can be ascribed to the hydrolysis of silane and to the performance of the following reaction with other silanols or isolated hydroxyl groups on the surface of the SF.

### 3.5. Soil Burial Testing

**Figure 5:**
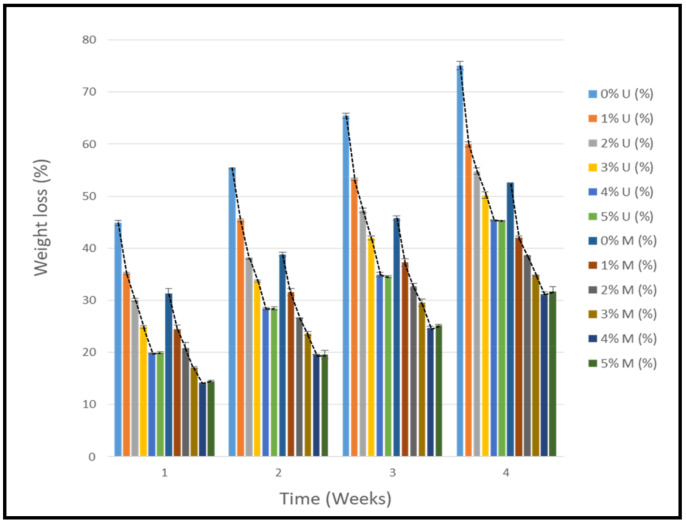
Weight loss (%) of seaweed/CNF composite film.

The objective of this investigation was to observe the potential of biodegradability of nanocomposites in soil burial tests. The major challenge of this test was to observe the weight loss accurately. The adherence of debris and soil was detached carefully from the sample specimen to obtain the true weight of the sample. Figure 5 and Table 2 show that the unmodified and silane modified seaweed/CNF composite films were degraded over time up to 30 days. Overall, each type of film degraded in the whole process. On the basis of observation, modified SF showed greater stability than unmodified SF. Seaweed nanocomposite films reinforced with CNF also lasted longer than control films with 0% CNF filler loading for both unmodified and modified SF. Increasing the CNF filler content eventually would increase the resistant of the SF to degrade over time. Figure 5 shows the weight loss percentages after 30 days of soil burial tests.

It can be clearly observed that the overall biodegradation test shows that silane modified films had a lower degradation rate compared to the unmodified films. In the final stages of testing, the unmodified and modified control films had weight loss values of 75.1% and 52.6%, respectively. The control modified films had around 22.5% lower weight loss compared to the unmodified films, which indicates better film stability with a lower rate of degradation. As for the effect of CNF incorporation into the film, the biodegradation rate of the biocomposite films was found to reduce significantly. During the first week of soil burial testing, the control film sample (0% CNF loading) had the highest degradation rate compared to incorporated films with CNF filler. The percentage weight loss for the unmodified and modified films was recorded at 44.9% and 31.3%, respectively. However, this value was decreased by the addition of CNF filler into the films. Incorporating 1% CNF into the seaweed films decreased the weight loss of the film to 35.2% and 24.5% for the unmodified and modified films, respectively.

Increasing the concentration of CNF filler would further decrease the percentage of weight loss for all the composite films, as shown by the trend line in Figure 5. On the fourth and final week of the experiment, the composite films with 4% CNF filler loading had the lowest percentages of weight loss at 45.4% and 31.2% for the unmodified and modified films, respectively. To put this into perspective, the composite films with 4% CNF loading had around 30% lower weight loss compared to control films with 0% CNF filler loading on the unmodified films and 20% lower for the modified films. However, increasing the CNF filler loading to 5% would not have decreased the degradation rate of the films, as the weight loss value of the films was slightly higher in composite films with 4% CNF loading.

The degradation of the unmodified control SF could undergo weight loss of up to 75.1% at 30 days due to its high moisture absorption property compared to the silane modified films, which had a lower rate of degradation. The hydrophilic nature of seaweed could be a factor in the weight loss of unmodified seaweed films due to the loss of low-molecular-weight compounds. Composites that are susceptible to water absorbtion tend to exhibit a higher rate of degradation [40]. Excess moisture uptake caused fractures on the surface of the seaweed composite films, exposing them to the environment and making them more vulnerable to microbial attack. The lower degradation rate for the modified film was due to the surface treatment of the films by the silane group, which enhanced the films’ moisture resistance. This effect can be clearly observed in Table 2 where the modified films show better films stability in terms of physical appearance, which indicates lower bacteria interaction compared to unmodified films that degrade rapidly. The hydroxyl group of the film membranes was greatly reduced by surface treatment with silane. When hydrolyzed silane solutions interact with SFs, the reactive silanol groups have a strong attraction to each other, creating the Si-O-Si connections; the same is true for the hydroxyl sites of fiber through hydrogen bonding. Thus, increasing the hydrophobicity of the films would eventually increase the resistance of the films toward biodegradation.

Due to the homogenous dispersion of CNF particles in the seaweed matrix, there is a strong interaction between the filler of the matrix and the CNF [41]. This interaction enhanced the stiffness of the seaweed films, resulting in a slower biodegradation rate of the seaweed films. The degradation of films becomes difficult as a result of the difficulty in breaking the strong bonds between the seaweed matrix and the CNF filler. Table 2 displays a series of images on the biodegradation test of the seaweed/CNF composite films over 30 days. All the films had relatively clean and smooth surfaces with a regular square shape before the soil burial test was performed. The seaweed composite films began to shrink and deteriorate from seven days onwards in compost soil. The surface texture and color tone of the seaweed/CNF composite films also changed. At the edges and on the surface of the composite films, cracks and voids were seen to develop over time. The composite films also became more darkened than the initial samples, especially the unmodified films, because of the higher hydrophilicity properties. The presence of microorganisms in nutritious dark garden soil can accelerate the biodegradation process [42]. The biodegradation rate of compost soil is higher than that of natural soil. Due to the increased nutrients offered by the compost environment, compost soil contains more microorganisms with faster metabolism rates than normal soil.

## 4. Conclusions

Seaweed composite films reinforced with varying amounts of CNF filler were successfully fabricated, modified, and characterized. Treatment of the silane surface and the incorporation of CNF filler improved the hydrophobicity and biodegradable properties of the seaweed composite films remarkably. Silane surface treatment with triethoxymethylsilane was shown to be successful on seaweed films. The modified seaweed films showed remarkable quality improvement by reducing the hydrophilicity nature of seaweed. Elimination of the hydroxyl group on the seaweed film membrane by polysiloxane provided a layer of protection for the films against moisture. Studies on water barrier properties (MAC, WS, WVP, and CA) showed a positive result on films that were moisture resistant. Films with hydrophobic properties are essential in packaging applications to withstand the original functionality of the film in wet and high-humidity conditions. The particle size analyzer confirmed that the diameter range of the kenaf CNF was observed to be between 6 and 12 nm. The result reveals that the particles were dispersed and distributed well without aggregation. The negative zeta potential value (−32.0 mV) indicates enough mutual repulsion for the filler to avoid the possibility of agglomeration during the fabrication of the biopolymer films. Seaweed and CNF fillers are biodegradable materials. Noticeable weight loss and changes in physical appearance were observed in the seaweed/CNF composite films during soil burial testing. The silane-modified films tended to have higher hydrophobicity properties and lower biodegradability compared to the unmodified films. The modified films had higher resistance to moisture, thus reducing the moisture interaction and bacterial activity on the films. The development of a seaweed/CNF composite film is a promising approach to diversifying and adding value the usage of seaweed and kenaf. The utilization of these materials also proved to be environmentally friendly and sustainable. In conclusion, the composite film is suitable to use as packaging material in various potential applications. Seaweed/CNF composite film could potentially be used as high-grade packaging material in the pharmaceutics, cosmetics, agriculture, and many other industries. The developed seaweed/CNF composite film is a promising approach to diversifying and adding value to the use of seaweed and kenaf as a high-grade packaging product.

## Figures and Tables

**Table 1:** Droplet images and contact angles (°) of seaweed/CNF composite film.

Filler Loading (%)	Contact Angle (θ)	
	Unmodified Film	Modified Film
**0**	* 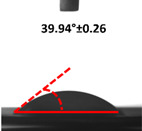 *	* 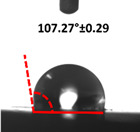 *
**1**	* 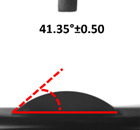 *	* 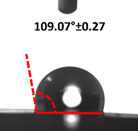 *
**2**	* 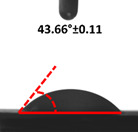 *	* 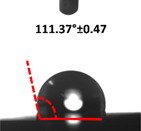 *
**3**	* 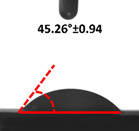 *	* 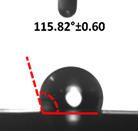 *
**4**	* 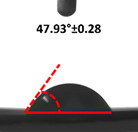 *	* 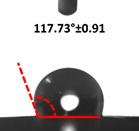 *
**5**	* 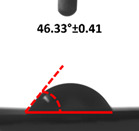 *	* 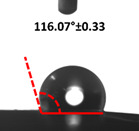 *

**Table 2:** Soil burial degradation of unmodified (a) and modified (b) seaweed/CNF composite film.

Weeks (a)	Unmodified Seaweed/CNF Composite Film					
	0%	1%	2%	3%	4%	5%
0	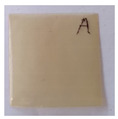	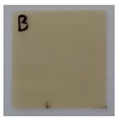	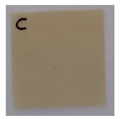	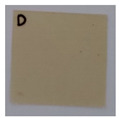	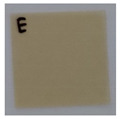	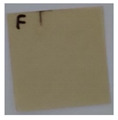
1	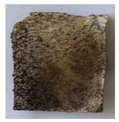	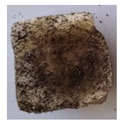	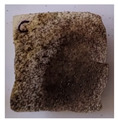	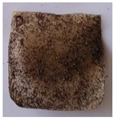	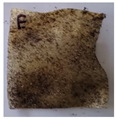	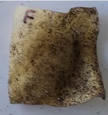
2	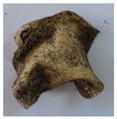	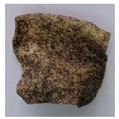	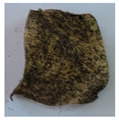	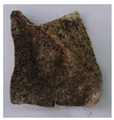	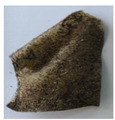	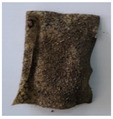
3	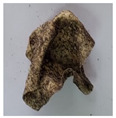	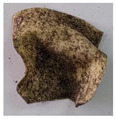	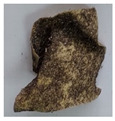	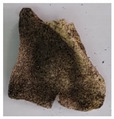	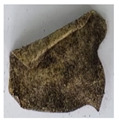	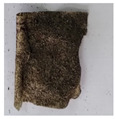
4	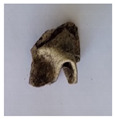	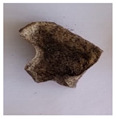	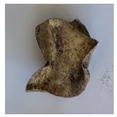	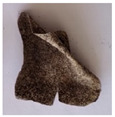	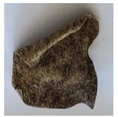	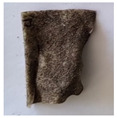
**Weeks** **(b)**	**Modified Seaweed/CNF Composite Film**					
	**0%**	**1%**	**2%**	**3%**	**4%**	**5%**
0	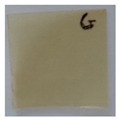	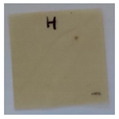	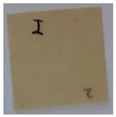	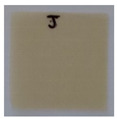	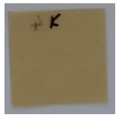	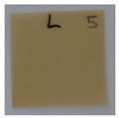
1	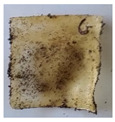	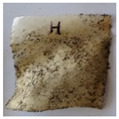	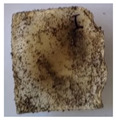	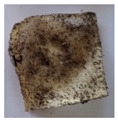	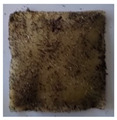	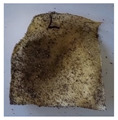
2	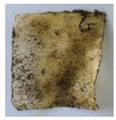	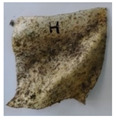	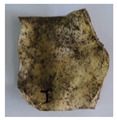	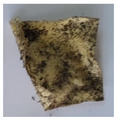	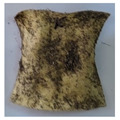	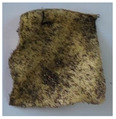
3	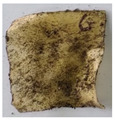	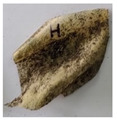	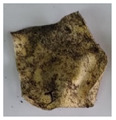	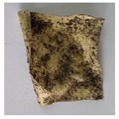	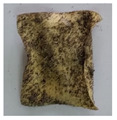	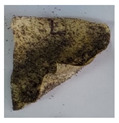
4	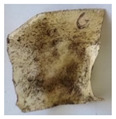	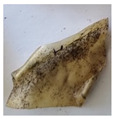	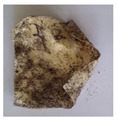	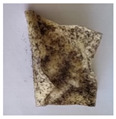	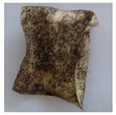	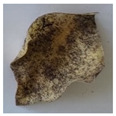

## Data Availability

Not applicable.
